# A secreted serine protease of *Paracoccidioides brasiliensis *and its interactions with fungal proteins

**DOI:** 10.1186/1471-2180-10-292

**Published:** 2010-11-16

**Authors:** Juliana A Parente, Sílvia M Salem-Izacc, Jaime M Santana, Maristela Pereira, Clayton L Borges, Alexandre M Bailão, Célia MA Soares

**Affiliations:** 1 Laboratório de Biologia Molecular, Instituto de Ciências Biológicas, Universidade Federal de Goiás, Goiânia, Goiás, Brazil; 2 Laboratório de Interação Parasito-Hospedeiro, Faculdade de Medicina, Universidade de Brasília, Brasília, DF

## Abstract

**Background:**

*Paracoccidioides brasiliensis *is a thermodimorphic fungus, the causative agent of paracoccidioidomycosis (PCM). Serine proteases are widely distributed and this class of peptidase has been related to pathogenesis and nitrogen starvation in pathogenic fungi.

**Results:**

A cDNA (*Pb*sp) encoding a secreted serine protease (*Pb*SP), was isolated from a cDNA library constructed with RNAs of fungal yeast cells recovered from liver of infected mice. Recombinant *Pb*SP was produced in *Escherichia coli*, and used to develop polyclonal antibodies that were able to detect a 66 kDa protein in the *P. brasiliensis *proteome. *In vitro *deglycosylation assays with endoglycosidase H demonstrated that *Pb*SP is a *N*-glycosylated molecule. The *Pb*sp transcript and the protein were induced during nitrogen starvation. The *Pb*sp transcript was also induced in yeast cells infecting murine macrophages. Interactions of *Pb*SP with *P. brasiliensis *proteins were evaluated by two-hybrid assay in the yeast *Saccharomyces cerevisiae*. *Pb*SP interacts with a peptidyl prolyl cis-trans isomerase, calnexin, HSP70 and a cell wall protein PWP2.

**Conclusions:**

A secreted subtilisin induced during nitrogen starvation was characterized indicating the possible role of this protein in the nitrogen acquisition. *Pb*SP interactions with other *P. brasiliensis *proteins were reported. Proteins interacting with *Pb*SP are related to folding process, protein trafficking and cytoskeleton reorganization.

## Background

Serine protease is a class of peptidases widely distributed in all domains of life that use a serine residue at the active site to cleave peptides [1]. Serine proteases are associated with virulence and nutrient cycling in many pathogens. In the human pathogen *Trichophyton rubrum *seven serine proteases genes were detected, two of them encoding products able to cleave keratin, suggesting the importance of these proteases in the invasion process in the human host [2]. Also, a secreted serine protease from *Microsporum canis *was described. A serine protease inhibitor, as well as a monoclonal antibody directed to the protein inhibited fungal adherence to reconstructed interfollicular feline epidermis [3]. In the entomophatogenic fungus *Magnaporthe grisea*, the SPM1 serine protease is positively regulated during nitrogen starvation condition. *M. grisea *mutant cells for the *spm*1 gene encoding for this serine protease present decreased sporulation and appressorial development as well as a greatly attenuated ability to cause disease [4]. Serine proteases play important role in nematophagous fungus during cuticle degradation. An alkaline serine protease was described as virulence factor in the nematophogous fungus *Hirsutella rhossiliensis *presenting higher protein expression level when nematode cuticle was used as the single source of nitrogen [5]. In the nematophagous fungus *Clonostachys rosea*, the disruption of the gene *pr*C encoding a subtilisin protease attenuated infection of the fungus to nematodes, indicating that this proteases acts as virulence factor [6].

*Paracoccidioides brasiliensis *is a thermally dimorphic fungus with a broad distribution in Latin America, the causative agent of the paracoccidioidomycosis. The infection is initiated by inhalation of airborne propagules of mycelia, which reach the lungs and differentiate into the yeast parasitic phase [7]. Few *P. brasiliensis *proteases have been characterized. Previous analysis of the ESTs in the transcriptome of mycelim and yeast cells revealed a total of 53 open reading frames (ORFs) encoding proteases in *P. brasiliensis*. The deduced amino acid sequences allowed the proteases to be classified in aspartyl, cysteine, metallo, serine proteases and proteasome subunits [8]. An extracellular subtilisin-like serine protease has been detected in the fungal yeast phase [9]. This protease is inhibited by PMSF (phenylmethyl-sulphonyl fluoride), mercury acetate and *p*-HMB (sodium 7-hydroxymercuribenzoate), allowing to classify the protein as a serine-thiol protease which was able to cleave, *in vitro*, murine laminin, human fibronectin, type IV-collagen and proteoglycans [10]. An aspartyl protease has been recently characterized in *P. brasiliensis*. The cDNA encoding the aspartyl protease (*Pb*sap) and the deduced amino acid sequence encoding this protease (*Pb*SAP) were identified and characterized. It was demonstrated that *Pb*SAP is a *N*-glycosylated molecule. This aspartyl protease was detected in the *P. brasiliensis *protein extract and culture supernatant, suggesting that *Pb*SAP is a secreted molecule. *Pb*SAP is also detected in the yeast cell wall by immunoelectron microscopy. Zymogram assays indicated the presence of aspartyl protease gelatinolytic activity in yeast cells and culture supernatant [11].

Transcriptome analysis of the *P. brasiliensis *yeast cells derived from infected mice [12] revealed a serine protease transcript positively regulated, wich was also induced in *P. brasiliensis *after incubation of yeast cells in human blood and plasma [13,14]. We analysed the effect of nitrogen deprivation on protein and transcript expression. Studies were also performed in order to characterize *Pb*SP interaction with other *P. brasiliensis *proteins. Our studies indicated the regulation of *Pb*SP by nitrogen availability and suggest additional roles of this serine protease in *P. brasiliensis*.

## Results

### Analysis of the cDNA and of the deduced protein sequence

The Additional file 1, presents the genomic and cDNA sequences encoding *Pb*SP. The cDNA sequence contains a 1491 bp open reading frame. The genomic sequence presents two introns and three exons. The deduced amino acid sequence presented 497 amino acids residues with a predicted molecular mass of 53 kDa and *pI *6.12. *Pb*SP homology analysis in MEROPS database reveals homology with serine proteases from S08 family of subtilases (data not shown). Analysis of the promoter region reveals a TATA box and a 5'-GATA-3' domain, putatively related to nitrogen metabolite regulation (NMR). Analysis of the deduced amino acid sequence revealed a 16 amino acid signal peptide, suggesting that *Pb*SP is a secreted molecule. Comparisons of the predicted protein sequence with well-known serine proteases allowed us to identify three conserved amino acids residues DHS that compose the catalytic triad of the subtilase family. Six N-glycosylation sites were also predicted at positions 76-79, 98-101, 160-163, 245-248, 287-290 and 450-453 in the deduced protein sequence (Additional file 1). The sequences of the serine proteases from *Ajellomyces dermatitidis *(GenBank EEQ89129), *Coccidioides posadasii *(GenBank EER27788) and *Aspergillus fumigatus *(GenBank XP_753718) showed the higher sequence identity to *Pb*SP (71%, 68% and 65%, respectively) (data not shown).

### Expression of *Pb*SP in *Escherichia coli *and antibody production

**Figure 1:**
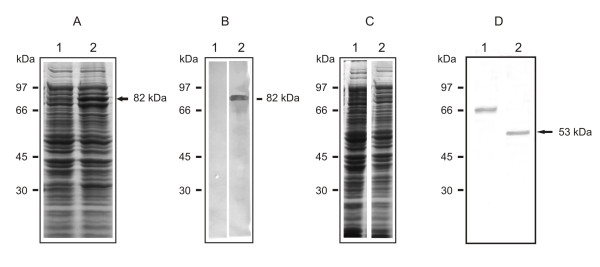
**Reactivity of the polyclonal antibodies anti-*Pb*SP and deglycosylation assay**. A: SDS-PAGE of *E. coli *extracts. The proteins were stained by Comassie blue. 1: *E. coli *protein extract; 2: *E. coli *protein extract obtained after 0.5 mM IPTG treatment. The arrow indicates the protein species corresponding to *Pb*SP fused to the GST protein. B: Western blot assay, the same extracts as in A reacted to: 1: Mice preimmune serum. 2: Polyclonal antibodies anti-*Pb*SP. C: SDS-PAGE of *P. brasiliensis *extracts 1: Total protein extract of yeast cells. 2: Total protein extract of yeast cells treated with endoglycosidase H for 16 h. D: Western blot using the polyclonal antibodies anti-*Pb*SP reacted with the protein extracts presented in C.

SDS-PAGE analysis of the bacterial transformants revealed that IPTG induced a dominant protein, migrating at 82 kDa (Figure 1A, lane 2). This dominant protein was absent in cells growing in the absence of IPTG (Figure 1A, lane 1). The size of the induced protein is in accordance to the expected size of the *Pb*SP fused to glutathione S-transferase (GST). The polyclonal antibody produced against *Pb*SP reacted with the recombinant protein in western blot analysis (Figure 1B, lane 2). No reaction was detected with preimmune serum (Figure 1B, lane 1). The polyclonal antibodies recognized a protein species of 66 kDa in *P. brasiliensis *proteome (Figure 1D, lane 1).

### Deglycosylation assays

The *Pb*SP molecular mass, as detected by western blot analysis (Figure 1D, lane 1) was higher in comparison to the value obtained to the deduced protein. The probable glycosylation of the molecule was analyzed by treating total protein extract of yeast cells with endoglycosidase H. Treatment with endoglycosidase H rendered a protein species of 53 kDa (Figure 1D, lane 2). The data support the inference that the 66 kDa protein in *P. brasliensis *yeast cells extract is the glycosylated form of the 53 kDa protein.

### Analysis of proteases expression during nitrogen starvation in *P. brasiliensis*

**Figure 2:**
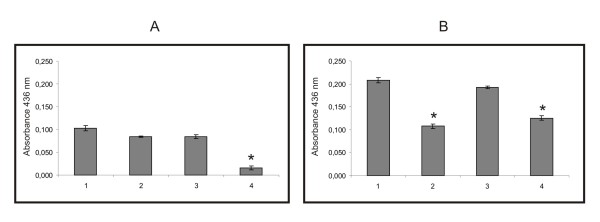
**Proteolytic activity of *P. brasiliensis *protein extracts**. Yeast cells were incubated in chemically defined MMcM medium with or without nitrogen sources (ammonium sulfate, asparagine and cystine) for 8 h. Protease activity was obtained by using azocasein assay. Activity was measured at 436 nm. A: Protease activity obtained in protein extracts of yeast cells incubated in MMcM medium. 1: without protease inhibitors; 2: with PMSF (1 mM); 3: with Pepstatin A (100 μM); 4: with EDTA (5 mM). B: Protease activity obtained in protein extracts of yeast cells incubated in MMcM medium without nitrogen sources. 1: without protease inhibitors; 2: with PMSF (1 mM); 3: with Pepstatin A (100 μM); 4: with EDTA (5 mM). Asterisk denotes values statistically different from control (*P *≤ 0.05).

The total proteases activity was analyzed in *P. brasiliensis *total protein extract during fungal nitrogen starvation. *P. brasiliensis *yeast cells were incubated in MMcM medium without nitrogen sources. Control reactions were performed. Protease activity was measured by using an azocasein assay in absence and presence of the protease inhibitors PMSF, Pepstatin A and EDTA. The total protease activity was higher in yeast cells extracts in the absence of nitrogen sources (Figure 2B, Bar 1). In the non-limiting nitrogen condition, a strong protease activity reduction was detected in the presence of EDTA (a metalloprotease inhibitor) (Figure 2A, Bar 4). In this condition the protease activity in the presence of PMSF or pepstatin was poorly reduced (Figure 2A, Bars 2 and 3, respectively). During nitrogen limiting condition the protease activity was strongly reduced in the presence of PMSF, a serine protease inhibitor (Figure 2B, Bar 2) and EDTA, a metalloprotease inhibitor (Figure 2B, Bar 4). It was observed no significant protease activity reduction in the presence of pepstatin A (Figure 2B, Bar 3).

**Figure 3:**
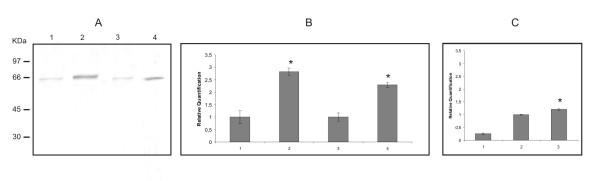
**Analysis of *Pb*sp and *Pb*SP expression during nitrogen starvation and during infection in murine macrophages**. A: Western blot assay using the polyclonal antibody anti-*Pb*SP of protein extracts of. 1: yeast cells cultured in MMcM medium; 2: yeast cells cultured in the same medium deprived of nitrogen; 3: culture supernatant of yeast cells in MMcM medium; 4: the same as in 3 in the absence of nitrogen. B: *Pb*sp quantification by Real Time PCR. RNAs obtained were used to obtain cDNAs used to perform *Pb*sp quantification. Reactions were performed in triplicate and normalized by using α-tubulin expression. 1: *Pb*sp relative quantification in yeast cells incubated in MMcM medium for 4 h; 2: *Pb*sp relative quantification in yeast cells incubated in MMcM medium without nitrogen sources for 4 h; 3: *Pb*sp relative quantification in yeast cells incubated in MMcM medium for 8 h; 4: *Pb*sp relative quantification in yeast cells incubated in MMcM medium without nitrogen sources for 8 h. C: *Pb*sp quantification by Real Time PCR. 1: *Pb*sp relative quantification in mycelium. 2: *Pb*sp relative quantification in yeast cells. 3: *Pb*sp relative quantification in yeast cells during infection in macrophages. Asterisk denotes values statistically different from control (*P *≤ 0.05).

The *Pb*SP expression was evaluated by western blot analysis after incubation of yeast cells in MMcM medium in the absence and in the presence of nitrogen sources. *Pb*SP expression was higher in yeast cells submitted to nitrogen starvation condition, both in total protein extract (Figure 3A, lane 2) and culture supernatant (Figure 3A, lane 4) in comparison to the *Pb*SP expression in the non-limiting nitrogen condition (Figures 3A, lanes 1 and 3).

### Analysis of *Pb*sp expression by quantitative real-time PCR

The *Pb*sp expression was evaluated by using real-time PCR in yeast cells submitted to nitrogen starvation. The *Pb*sp expression was strongly induced during limiting nitrogen condition in 4 and 8 h (Figure 3B, Bars 2 and 4), compared to the non-limiting condition (Figure 3B, Bars 1 and 3). The *Pb*sp expression was also evaluated in mycelium, yeast cells and yeast cells infecting macrophages. The results are presented in Figure 3C. The *Pb*sp expression in mycelium is strongly reduced (Figure 3C, Bar 1) compared to the *Pb*sp expression in yeast cells (Figure 3C, Bar 2). There is an increased *Pb*sp expression in yeast cells infecting macrophages (Figure 3C, Bar 3).

### Interaction of serine protease with other *P. brasiliensis *proteins

**Figure 4:**
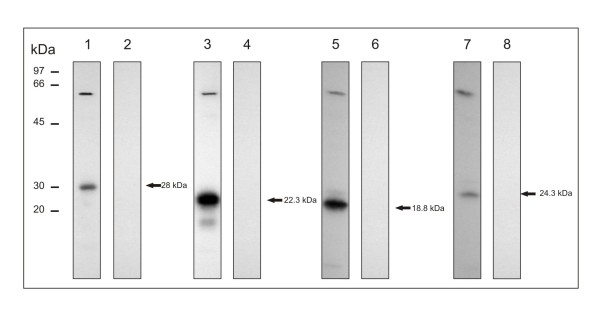
**Co-immunoprecipitation of *P. brasiliensis *proteins putatively interacting with *Pb*SP**. *Pb*SP and the proteins found interacting with this protease in the two-hybrid assay were in vitro synthesized and labeled with ^35^S methionine. The translated serine protease fused to c-myc epitope (c-myc-SP) and the translated proteins fused to hemaglutinin epitope (HA-Prey) were mixed and the mixture was incubated with protein A agarose beads and the monoclonal antibody anti-c-myc. The proteins were separated by SDS-PAGE. The gel was fixed, dried under vacuum and autoradiography was obtained. 1: Peptidyl prolyl cis-trans isomerase; 3:Calnexin; 5: HSP70; 7: Periodic tryptophan protein (PWP2). Negative controls for each reaction were performed and are shown in the lanes 2, 4, 6 and 8, respectively.

The interaction of *Pb*SP with other *P. brasiliensis *proteins was evaluated by two-hybrid system in *S. cerevisiae*. The proteins identified interacting with *Pb*SP are described in Table 1. It was detected homologues of FKBP-peptidyl prolyl cis-trans isomerase, calnexin, HSP70 and a possible cytoskeleton associated periodic tryptophan protein. Protein interactions were confirmed by co-immunoprecipitation assays and are shown in Figure 4.

**Table 1:** P. brasiliensis proteins which interact with PbSP as determined by two-hybrid assay in S. cerevisiae.

Gene Product	Best hit	e-value	Number of obtained clones
FKPB-type peptidyl prolyl cis trans isomerase	*Aspergillus clavatus *XP_001274819	2e^-25^	4
Calnexin	*P. brasiliensis *ABB80132	2e^-28^	2
Mitochondrial 70 kDa heat shock protein	*P. brasiliensis *AAP05987	6e^-83^	2

Periodic tryptophan protein PWP2	*Ajellomyces capsulatus *XP_001543414	2e^-30^	1

## Discussion

The *P. brasiliensis *serine protease cDNA/gene here characterized encode a protein with a N-terminal 16 amino acids with the characteristic of a leader peptide. The protein sequence corresponding to the mature *Pb*SP shows high similarity with serine proteases sequences from other fungi. Analysis of the promoter region revealed the presence of a nitrogen metabolite repression (NMR) region binding protein, responsible for positive regulation of genes in response to nitrogen metabolite presence such as AreA proteins in *Aspergillus nidulans *[15] and Nit2 protein in *Neurospora crassa *[16]. The data suggest that *Pb*SP could be a molecule regulated by the nitrogen metabolite presence.

The recombinant *Pb*SP was obtained fused to GST, exhibiting a molecule of 82 kDa. By using the recombinant protein, polyclonal antibodies were obtained in mice. The serum, specifically, recognized the recombinant protein as well as a protein species of 66 kDa in *P. brasiliensis *yeast cells extract. Treatment of fungal protein extracts with endoglycosidase H resulted in a 53 kDa protein species, corresponding to the *Pb*SP *in silico *deduced molecular mass. The data suggest that the 13 kDa additional in the 66 kDa species is due to N-glycosylation.

Total protease activity was evaluated during fungal nitrogen starvation by incubating yeast cells in chemically defined medium in the presence and absence of nitrogen sources. Protease activity was higher in the absence of nitrogen sources. Protease activity was also evaluated in the presence of specific inhibitor to serine, aspartyl and metalloprotease. In the presence of nitrogen sources, the most reduced activity was detected in the presence of EDTA indicating that metalloproteases have higher activity in nitrogen non-limiting condition. In nitrogen-limiting conditions, the protease activity was reduced in the presence of PMSF and EDTA, suggesting that serine proteases activity is higher in the nitrogen starvation condition.

In accordance to the Western blot and qRT-PCR results, *Pb*SP and *Pb*sp expression levels were higher during nitrogen starvation. *Pb*SP was detected by Western blot in the yeast cell culture supernatant, suggesting this is a secreted protease and could be related to the nitrogen starvation response in *P. brasiliensis*. The nitrogen starvation response can be important in human pathogens since neutrophil phagosome presents low nitrogen concentration. In this way, the *S. cerevisiae *and *Candida albicans *transcriptional profiles during neutrophil internalization are most similar to that of amino acid deprivation [17]. Similarly, a subtilisin like serine protease from *Mycobacterium tuberculosis *is described as a cell wall-associated protein and is induced during infection of macrophages [18].

Serine protease can be relevant during the infectious process. We demonstrated increased *Pb*sp expression in *P. brasiliensis *yeast cells infecting macrophages. The serine protease importance during infection was also reported to the pathogenic dermatophyte *Arthroderma benhamiae *since these proteases were positively regulated during experimental infection in guinea pig as demonstrated by using cDNA microarray analysis [19]. In the fungus *Histoplasma capsulatum*, a range of proteins associated to pathogenesis are secreted, including a serine protease, detected in vesicles of the parasitic yeast phase [20]. Also, *Candida *spp. isolated from gingival erythema are able to secret serine proteases that may be involved in the initial colonization events since the pre-treatment of *Candida *spp. cells with the serine protease inhibitor PMSF diminished the *Candida *spp. interaction with epithelial cells [21].

Two hybrid assays were performed to detect *P. brasiliensis *proteins interactions with *Pb*SP. *Pb*SP interacts with proteins presumably related to protein processing such as FKBP-peptidyl prolyl cis-trans isomerase, calnexin and HSP70. The *Pb*SP interaction with these proteins could be related to protein processing such as retention of incorrectly folded proteins [22], trafficking of serine protease into and through the compartments in the cell [23] and acceleration of folding process [24]. Glycosylation has been associated to many processes such as folding, transport, secretion and degradation of the proteins containing the glycan chains. These processes are mediated by proteins that recognize these glycan chains, such as lectin-chaperones and calnexin and occurs in the endoplasic reticulum [25]. The demonstrated interaction of *Pb*SP with calnexin can be related to the protein N-glycan chains. Work will focus in this subject. Calnexin is also related to protein secretion [26]. The detection of *Pb*SP as a secreted molecule could reinforce its association with calnexin, as demonstrated. The PWP2 protein also interacts with serine protease. PWP2, in the cytoplasm, may be associated to the cytoskeleton since *S. cerevisiae *strains presenting depletion of the PWP2 gene are defective in the hydrolysis of the septal junction between mother and daughter cells and cell growth [27]. Further analyses are required to confirm the relevance of the *Pb*SP interaction with these proteins.

## Conclusions

In the present work a serine protease was characterized. This protease is a N-glycosylated molecule detected by immunoassay in *P. brasiliensis *cellular proteins and culture supernatant. This secreted protease and the cognate transcript were induced by nitrogen starvation indicating its possible role in the nitrogen acquisition. Protein interactions with serine protease were firstly reported. *Pb*SP interacts with proteins related to protein folding such as calnexin and FKBP-peptidyl prolyl cis-trans isomerases. *Pb*SP interactions with HSP70 and with a PWP protein were also detected. The function of the interactions with *Pb*SP molecules are possibly related to acceleration and quality control of *Pb*SP folding and trafficking to compartments in the cell. Interaction with a possible cytoskeleton protein was also reported, suggesting that the *Pb*SP could be associated to different proteins in many subcellular localizations, playing role in a range of processes.

## Methods

### *P. brasiliensis *isolate growth conditions

*P. brasiliensis *isolate *Pb*01 (ATCC MYA-826) was maintained at 36°C in Fava-Netto's medium [1% (w/v) peptone; 0.5% (w/v) yeast extract; 0.3% (w/v) proteose peptone; 0.5% (w/v) beef extract; 0.5% (w/v) NaCl; 1.2% (w/v) agar, pH 7.2]. For nitrogen starvation experiments, *P. brasiliensis *yeast cells (10^6 ^cells/mL) were cultured in liquid MMcM minimal medium [1% (w/v) glucose, 11 mM KH_2_PO_4_, 4.15 mM MgSO_4_·7H_2_O, 20 μM CaCl_2_·2H_2_O, 15.14 mM NH_4_SO_4_, 0.02% (w/v) L-asparagine, 0.002% (w/v) L-cystine, 1% (v/v) vitamin solution - contaning thiamine hydrochloride, niacin, calcium pantothenate, inositol, biotin, riboflavin, folic acid, choline chloride, pyridoxine hydrochloride - and 0.1% (v/v) trace element supplement - containing H_3_B0_3_, CuSO_4·_5H_2_0, Fe(NH_4_)2(SO_4_)_2_·6H_2_0, MnSO_4_·4H_2_0, (NH_4_)6Mo_7_0_24_·4H_2_0, ZnSO_4_·7H_2_0,] [28] without ammonium sulfate, asparagine and cystine during 4 and 8 h. Control condition was performed by incubation of yeast cells in liquid MMcM minimal medium containing the nitrogen sources ammonium sulfate, asparagine and cystine during 4 and 8 h. For murine macrophages infection, *P. brasiliensis *yeast cells were grown in RPMI 1640 medium (Biowhittaker, Walkersville, Md.).

### Obtaining the *P. brasiliensis *serine protease cDNA and bioinformatics analysis

A complete cDNA encoding a *P. brasiliensis *homologue of the serine protease was obtained from a cDNA library of yeast cells recovered from liver of infected mice [12]. The cDNA was sequenced on both strands by using the MegaBACE 1000 DNA sequencer (GE Healthcare) and the predicted amino acid sequence was obtained. The protease classification was performed by using the MEROPS database http://merops.sanger.ac.uk. The entire nucleotide sequence, *Pb*sp, and the predicted amino acid sequence, *Pb*SP, have been submitted to the GenBank database under accession number AY319300.

The National Center for Biotechnology Information (NCBI) BLASTp algorithm http://www.ncbi.nlm.nih.gov was used to search in the non-redundant database for proteins with sequence similarities to the translated full-length *Pb*SP cDNA. The ScanProsite algorithms http://ca.expasy.org/tools/scanprosite/ were used to search for motifs and conserved domains in the deduced protein. The presence of signal peptide was identified by using the SignalP program http://www.cbs.dtu.dk/services/SignalP/, while the prediction of cellular localization was performed by using the PSORT II algorithm http://psort.ims.u-tokyo.ac.jp/form2.html. The complete genomic sequence of *Pb*sp was obtained in the *P. brasiliensis *genomic database http://www.broad.mit.edu/science/projects/msc/data-release-summary and the promotor region was analyzed by using the Promotor scan algorithms http://www-bimas.cit.nih.gov/cgi-bin/molbio/proscan.

### Cloning of *Pb*SP cDNA into expression vector

Oligonucleotide primers were designed to amplify the complete cDNA encoding the *Pb*SP. The nucleotide sequence of the sense and antisense primers were 5'-TCTGGATCCATGAAAGGCCTCTTCGC-3' and 5'-ACACTCGAGTCCAGAGATGAAAGCGTT-3', containing *Bam*HI and *Xho*I restriction sites, respectively (underlined). The amplification parameters were as following: 94°C for 2 min, followed by 30 cycles of denaturation at 94°C for 20 s, annealing at 50°C for 20 s, and extension at 72°C for 2 min; final extension was at 72°C for 5 min. The PCR product was electrophoresed and a 1491 bp amplicon was gel excised and cloned into the pGEX-4T-3 expression vector (GE Healthcare). The recombinant plasmid was used to transform the *E. coli *strain C43(DE3) competent cells by using the heat shock method [29]. Ampicilin-resistant transformants were cultured, and plasmid DNA was analyzed by PCR and DNA sequencing, as described above.

### Heterologous expression of *Pb*SP and antibody production

The protein heterologous expression was performed as described [30] with modifications. Cultures of transformed *E. coli *containing pGEX-4T-3 cloned with *Pb*sp were grown in Luria-Bertani (LB) medium supplemented with 100 μg/ml of ampicillin, at 37°C. As the cells reach the log phase (A_600 _0.6), IPTG (isopropyl-β-D-thiogalactopyranoside) was added to the growing culture to a final concentration of 0.5 mM to induce protein expression. After 2 h incubation, the bacterial cells were harvested by centrifugation at 5.000 *g *and ressuspended in phosphate saline buffer (PBS) 1×. *E. coli *cells transformed with pGEX-4T-3 and *E. coli *were used as controls. The cell extracts ressuspended in PBS 1× were electrophoresed on a 10% SDS-PAGE, followed by Coomassie brilliant blue staining. The protein species corresponding to *Pb*SP fused to glutathione S transferase (*Pb*SP-GST) was excised from the gel and 200 μg of the material was used to inoculate mice through subcutaneous injection. Animal was boosted three times, at 2 weeks intervals, with the same amount of antigen. The obtained serum, containing anti-*Pb*SP polyclonal antibodies was sampled and stored at -20°C. Preimmune serum was obtained.

### Obtaining cell extracts and secreted proteins of *P. brasiliensis*

Total protein extracts from *P. brasiliensis *yeast cells was obtained [31]. Briefly, frozen cells (3 g) were disrupted by complete grinding with a mortar and pestle in buffer (20 mM Tris-HCl, pH 8.8, 2 mM CaCl_2_) without protease inhibitors. The mixture was centrifuged at 15,000 *g *at 4°C, for 20 min; the supernatant was sampled, and stored at -80°C. Culture supernatant of yeast cells was obtained after 8 h incubation in liquid MMcM minimal medium. The cells were separated by centrifugation at 5,000 *g *for 15 min and the supernatant was filtered in a 0.22 μm filter. The culture supernatants were dialyzed with water during 4 h at 4 ºC. Secreted protein fraction was concentrated with ice-cold acetone (v/v) during 16 h, centrifugated at 15,000 *g *for 15 min and the pellet was washed with 70% (v/v) ice-cold acetone. Each 50 mL of culture supernatant was concentrated to 500 μL in Tris-HCl 25 mM pH 7.0. Protein concentration of all the samples was measured by using Bradford reagent (Sigma Aldrich) using BSA as standard.

### Western blot analysis

SDS-polyacrylamide gel electrophoresis (SDS-PAGE) was performed as described [32]. Proteins were electroblotted to a nylon membrane and transfer was checked by Pounce S staining. The membrane was blocked with 5% (w/v) non-fat dried milk in PBS 1× (pH 7.4). Serine protease was detected with the polyclonal antibody to the recombinant protein. After reaction with alkaline phosphatase anti-mouse immunoglobulin G (IgG), the reaction was developed with 5-bromo-4-cloro-3-indolylphosphate-nitroblue tetrazolium (BCIP-NBT). Negative controls were obtained with preimmune serum.

### Glycosylation analysis

The glycosylation analysis was performed as described [11]. Total protein extract from yeast cells was incubated with recombinant endoglycosidase H (Endo H) from *Streptomyces plicatus *(Sigma-Aldrich), for 16 h at 37°C. The reaction mixture (100 μl) contained 30 μg of the protein extract and 27 mU Endo H in 60 mM sodium acetate buffer pH 5.8. Samples were analyzed by western-blot.

### Azocasein assay

The azocasein assays were performed as described [33] with modifications. Azocasein was diluted to 5 mg/mL in buffer containing 25 mM Tris-HCl, 200 mM NaCl, 25 mM CaCl_2, _0.05% (v/v) Nonidet P-40 and 0.01% (w/v) NaN_3_. A total of 150 μg of *P. brasiliensis *total protein extract were used in each assay, performed in triplicate. Azocasein assay was performed in presence and absense of the specific protease inhibitors: 1 mM PMSF (serine protease inhibitor), 100 μM Pepstatin A (aspartyl protease inhibitor) and 5 mM EDTA (metalloprotease inhibitor). Proteinase K (Sigma Aldrich) was used as positive control. Azocasein assays with significant differences were determined by statistical analysis by using *t *test. *P *values of 0.05 or less were considered statistically significant.

### Preparation and infection of murine macrophages

Bone marrow-derived macrophages were obtained by flushing the femurs of 4-12 weeks old female C57BL/6 mice. The cells were cultured as described [34]. Briefly, the obtained cells were cultured for 8 days. The non-adherent cells were discarded and the adherent cells were washed twice with 10 mL of Hank's Balanced Salt Solution (HBSS). After cells treatment with 10 ug/mL of dispase (Invitrogen) in HBSS (37°C for 5 min), macrophages were removed using a cell scraper and washed in HBSS. Cells were resuspended in RPMI 1640 (10^6 ^cells/mL). For infection experiments, 10^7 ^*P. brasiliensis *yeast cells were added to 2 mL of macrophage suspension and co-cultivated for 24 h (37°C in 6% CO2). The wells were washed twice with HBSS to remove unattached yeast forms. RNA from infected murine macrophages was extracted by using Trizol reagent. RNAs from uninfected macrophages and from *P. brasiliensis *yeast cells cultured in RPMI 1640 medium were obtained as control.

### Quantitative real-time PCR

RNA samples were reverse transcribed by using the High Capacity RNA-to-cDNA kit (Applied Biosystems, Foster City, CA). The cDNA samples were diluted 1:2 in water, and qRT-PCR was performed using SYBR green PCR master mix (Applied Biosystems, Foster City, CA) in the Applied Biosystems Step One Plus PCR System (Applied Biosystems Inc.). qRT-PCR was performed in triplicate for each cDNA sample. The specificity of each primer pair for the target cDNA was confirmed by the visualization of a single PCR product in agarose gel electrophoresis. The primers and sequences were used as follows: serine-sense, 5'-GGCCTCTCCACACGTTGCTG-3'; serine-antisense 5'-GTTCCAGATAAGAACGTTAGC-3' and α-tubulin primers: tubulin-sense, 5'-ACAGTGCTTGGGAACTATACC-3'; tubulin-antisense, 5'-GGACATATTTGCCACTGCCA-3'. The annealing temperature for serine and tubulin primers was 60°C. The standard curves were generated by using the cDNAs serially diluted 1:5 from the original dilution. The relative expression levels of genes of interest were calculated using the standard curve method for relative quantification [35]. Statistical analysis was calculated by using *t *test. *P *values of 0.05 or less were considered statistically significant.

### Interaction of *Pb*SP with *P. brasiliensis *proteins as determined by Two-Hybrid assay

Oligonucleotides were designed to clone the complete cDNA encoding the *Pb*SP in the pGBK-T7 (Clontech Laboratories, Inc) expression vector. The nucleotide sequence of the sense and antisense primers were 5'-CATATGATGAAAGGCCTCTTCGCCT-3' and 5'-CTGCAGTTAAGAGATGAAAGCGTTCTTG-3', contained engineered *Nde*I and *Pst*I restriction sites, respectively (underlined). The pGBK-T7 contains the TRP1 gene which allows the selection in minimal medium without tryptophan and a GAL4 DNA-binding domain. The cloned product was used to transform a *S. cerevisiae *strain Y187 (ΔTRP1). A cDNA library was constructed with RNA from *P. brasiliensis *yeast cells and cloned in the expression vector pGADT7-Rec by using the Matchmaker™Library Construction & Screening (Clontech Laboratories, Inc) [36]. The pGADT7-Rec vector contains LEU2 gene, allowing the selection in minimal medium without leucine and a GAL4 DNA-activation domain. The cloned products were transformed in *S. cerevisiae *strain AH109 (Δ LEU2). The Y187 strain containing pGBK-T7-*Pb*SP was used to screen the pGADT7-Rec library transformed in AH109 strain by yeast mating. The positive interactions activate the transcription of ADE2, HIS3 and MEL1 genes, which allows the selection in minimal medium without tryptophan, leucine, adenine and histidine. Minimal medium without these amino acids and containing X-alpha-GAL also confirms the activation of the transcription of the MEL1 gene. The *Pb*SP baited clones were amplified by using AD-LD 5' (5'-CTATTCGATGATGAAGATACCCCACCAAACCC-3') and AD-LD 3' (5'-GTGAACTTGCGGGGTTTTTCAGTATCTACGATT-3') oligonucleotides for pGADT7-Rec and sequenced as described above. The positive interactions were confirmed by using the *in vitro *translation system TNT^® ^T7 Coupled Reticulocyte Lysate Systems (Promega Corporation) with S^35 ^methionine and coimmunoprecipitation of the translated proteins (Matchmaker™ Co-IP Kit, Clontech Laboratories, Inc). Briefly, the translated serine protease fused to c-myc epitope (c-myc-SP) and the translated proteins fused to hemaglutinin epitope (HA-Prey) were mixed at 25°C for 1 h. The mixture was incubated with protein A Agarose beads and with the monoclonal c-myc antibody in PBS at 25°C for 1 h. After washing, the beads containing proteins were resuspended in SDS-loading buffer [50 mM Tris-HCl, pH 6.8; 100 mM dithiothreitol, 2% (w/v) SDS; 0.1% (w/v) bromophenol blue; 10% (v/v) glycerol], followed by boiling at 80°C for 5 min. The proteins were separated on a SDS-PAGE 4-12% linear gradient. The gel was fixed with 20% (v/v) ethanol and 10% (v/v) acetic acid for 30 min, and incubated in 20 mL of fluorographic reagent NAMP 100 (Amplify Fluorographic Reagent - GE Healthcare^®^). The gels were dried at 80°C for 90 min under vacuum and autoradiography was obtained. Controls were performed. Each assay was repeated three times with a different batch of *in vitro *translated product to confirm the results.

## Authors' contributions

JAP contributed to the cloning and heterologous expression experiments, microrganisms cultures, glycosylation experiment, western blot analysis, protease activity and contributed to two-hybrid library construction. SMSI and CLB contributed in the two-hybrid library construction and co-immunoprecipitation experiments. JMS performed the polyclonal antibodies production. MP contributed to the data analysis. AMB performed the macrophage preparation and contributed to the real time PCR experiments. CMAS designed the project, contributed to the data analysis and to the preparation of the manuscript. All authors read and approved the final manuscript.

## Supplementary Material

Additional file 1 **The cDNA and the genomic sequences encoding the serine protease (*Pb*SP) of *P. brasiliensis***. The nucleotide and amino acid positions are marked on the left side. Lower case letters represent the untranslated 5' region. Bold letters in nucleotide sequence represent the start and stop codons. Two introns were found in the genomic sequence and are shown in italic. Three conserved residues (marked with arrows) of amino acids (asparagine - D; histidine - H and serine - S) belonging to the active site of serine proteases from the subtilase family S08 are evidenced. Six putative N-glycosylation sites are marked in bold letters. A signal peptide formed by the first 16 amino acids is underlined. The TATA box in the promoter region is evidenced by white letters. A GATA binding region of the transcription factor AreA was found and is evidenced by a white box. Click here for file
