# Supplementary figures and images for: A secreted serine protease of Paracoccidioides brasiliensis and its interactions with fungal proteins
Source: BMC Microbiol. 2010 Nov 16;10:292. doi: 10.1186/1471-2180-10-292 (PMC3000847; doi:10.1186/1471-2180-10-292)

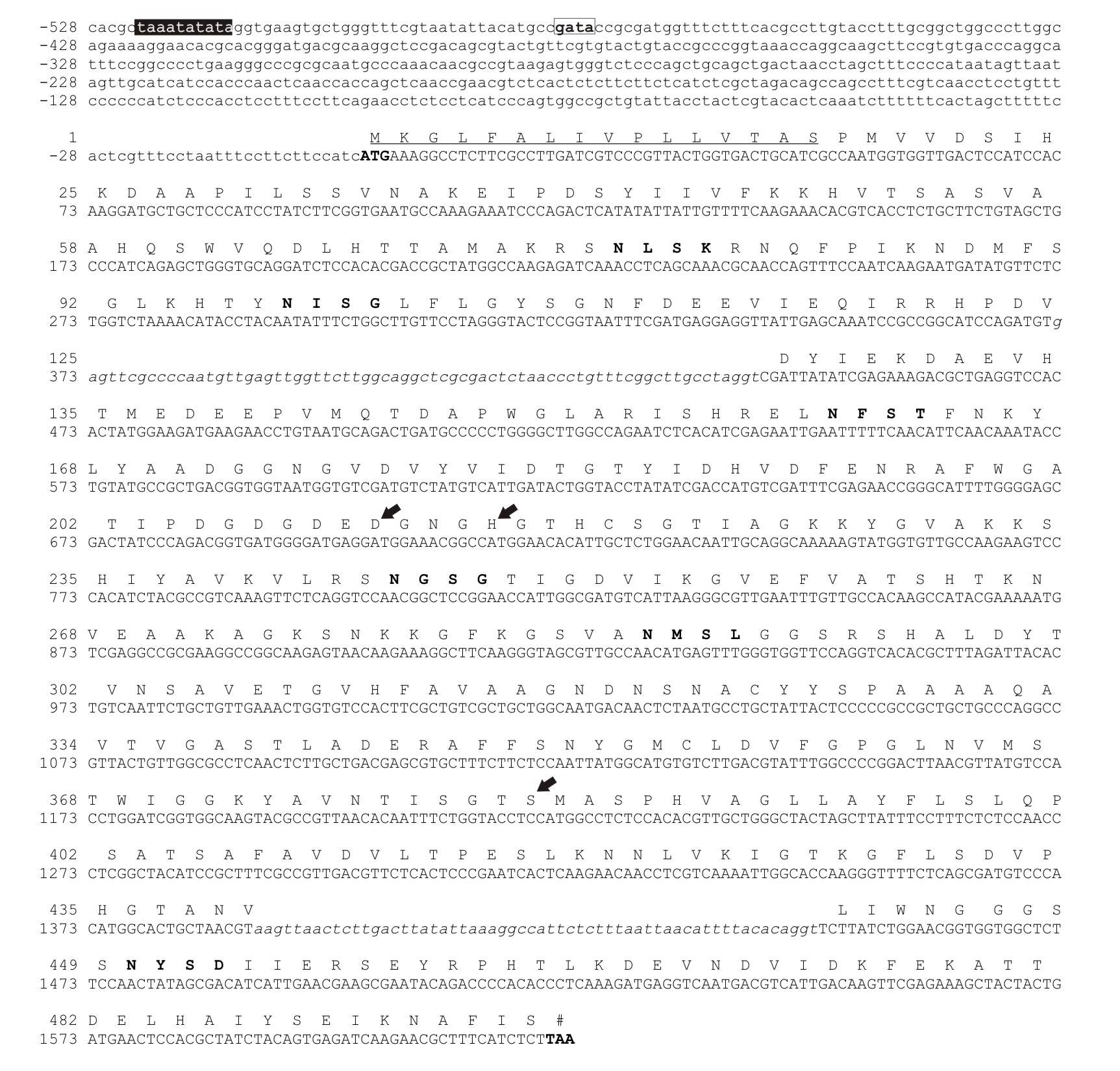

Supplement: Additional file 1 — The cDNA and the genomic sequences encoding the serine protease (PbSP) of P. brasiliensis. The nucleotide and amino acid positions are marked on the left side. Lower case letters represent the untranslated 5' region. Bold letters in nucleotide sequence represent the start and stop codons. Two introns were found in the genomic sequence and are shown in italic. Three conserved residues (marked with arrows) of amino acids (asparagine - D; histidine - H and serine - S) belonging to the active site of serine proteases from the subtilase family S08 are evidenced. Six putative N-glycosylation sites are marked in bold letters. A signal peptide formed by the first 16 amino acids is underlined. The TATA box in the promoter region is evidenced by white letters. A GATA binding region of the transcription factor AreA was found and is evidenced by a white box. [file 1471-2180-10-292-S1.JPEG]
